# Interactions of *Vallisneria natans* and Iron-Oxidizing Bacteria Enhance Iron-Bound Phosphorus Formation in Eutrophic Lake Sediments

**DOI:** 10.3390/microorganisms10020413

**Published:** 2022-02-11

**Authors:** Juanjuan Wang, Mingming Gao, Yanju Yang, Shipeng Lu, Guiliang Wang, Xiaoqing Qian

**Affiliations:** 1 College of Environmental Science and Engineering, Yangzhou University, Yangzhou 225127, China; wangjuanjuan@yzu.edu.cn (J.W.); gm15161936801@163.com (M.G.); 006468@yzu.edu.cn (Y.Y.); 2 Institute of Botany, Jiangsu Province and Chinese Academy of Sciences, Nanjing 210014, China; lvshipeng@cnbg.net; 3 Jiangsu Collaborative Innovation Center for Solid Organic Waste Resource Utilization, Nanjing 210014, China

**Keywords:** bacterial community structure, bacterially mediated iron oxidation, eutrophic lake sediments, iron-bound phosphorus, *Vallisneria natans*

## Abstract

Submerged macrophyte restoration and in situ phosphorus (P) passivation are effective methods for the control of internal P loading from sediments. This study explored the synergistic effects of *Vallisneria natans* and iron (Fe)-oxidizing bacteria (IOB) on internal P loading from eutrophic freshwater lake sediments by taking into account Fe-bound P (FeP) formation and associated bacterial community structures. Sediment samples were prepared in glass tanks under four treatments, namely no *V. natans* planting or IOB inoculation (control), planting *V. natans* without IOB inoculation (Va), planting *V. natans* with IOB inoculation (Va-IOB), and planting *V. natans* with autoclaved IOB inoculation (Va-IOB[A]). Compared with the control, all three treatments with *V. natans* (Va, Va-IOB, and Va-IOB[A]) had significantly decreased organic matter contents and increased redox potential in sediments (*p* < 0.05), at the rapid growth and mature stages of *V. natans*. Planting *V. natans* with and without IOB inoculation also decreased the total P (TP) and Fe–P concentrations in sediments. Conversely, Fe^3+^ concentrations, Fe^3+^/Fe^2+^ ratios, and the proportions of Fe–P in TP all increased in sediments planted with *V. natans*, especially under the Va-IOB treatment (*p* < 0.05). Furthermore, bacterial community diversity increased in sediments due to the presence of *V. natans*. The relative abundances of IOB (including *Acidovorax* and *Chlorobium*) increased from the transplanting to the rapid growth stage of *V. natans* and then decreased afterwards. In the later stages, the relative abundances of IOB and their ratios to Fe-reducing bacteria were the highest under the Va-IOB treatment. Accordingly, synergistic interactions between *V. natans* and IOB could enhance Fe–P formation and reduce TP concentrations in eutrophic lake sediments by altering sediment physicochemical properties and Fe oxidation-related bacterial community structures.

## 1. Introduction

Eutrophication of freshwater bodies is caused primarily by excess nutrient inputs, especially phosphorus (P). P is considered the key limiting nutrient in the water eutrophication process, with nitrogen (N) being the second most important element [1]. Controlling external P input has been proposed as one of the main strategies of mitigating eutrophication in lakes [2]. However, P release from surface sediments often leads to delayed recovery of freshwater from eutrophication following the reduction of external P input [3]. Consequently, the control of internal P loading from sediments has increasingly attracted the attention of researchers and stakeholders in eutrophication management in freshwater ecosystems.

Among the numerous water remediation technologies, submerged macrophyte restoration and in situ P passivation are two effective methods of controlling internal P loading. For example, the common macrophyte *Vallisneria natans* (eelgrass) can absorb P and N from the water column and surface sediments [4,5,6]. In addition, submerged macrophytes increase sediment redox potential (Eh), which in turn enhances P retention in sediments and reduces its release into the water column [7]. Submerged macrophytes are distributed extensively in freshwater environments and can be obtained relatively easily; consequently, they represent ideal natural materials for controlling internal P loading from sediments. In addition, the efficiency of submerged macrophytes in controlling internal P loading may be enhanced by integration with in situ P passivation [8,9].

The adsorption technique has shown tremendous potential for in situ P passivation in sediments, with widespread use and simple operation [6,10]. Adding absorbents such as iron (Fe) compounds could enhance P retention in the sediment and limit the increase in dissolved P concentration in the water column. Li et al. [11] found that the addition of ferric chloride could convert P into more stable organic forms and could effectively inhibit the release of internal P from the sediment. Furthermore, Fe-modified calcite, as an adsorption material, could remove 91.7% of P from the water column and maintain 90.8% of sediment P in a stable state, reducing the potential of P release from sediments [12]. In situ P passivation techniques, however, have some limitations. For example, large-scale applications of adsorbents for passivizing P in sediments often have high manufacturing and transportation costs.

Microbial remediation of polluted environments has broad application prospects considering its simple operation, low cost, high efficiency, and minimal impacts on the environment [13,14]. An improved in situ P passivation method involves inoculation of sediment with functional microorganisms that participate in Fe redox transformation; in this way, Fe redox speciation can be used to regulate P transfer between the sediment and water column [7]. In shallow eutrophic lakes, P retention in sediments has been reported to be controlled mainly by the oxidation of Fe^2+^ [15]. Iron-oxidizing bacteria (IOB) can oxidize Fe^2+^ into Fe^3+^ and form Fe oxides such as ferric hydroxide (FeOOH) colloids. The FeOOH colloids have a high PO_4_^3−^ absorption capacity and promote the formation of Fe-bound P (Fe–P) [16].

Generally, Fe–P refers to the P adsorbed on the surface of FeOOH by physical or chemical actions [17], and its release process is influenced by multiple environmental factors, such as pH and Eh [18,19]. When Eh decreases, Fe^3+^ is reduced to Fe^2+^, leading to FeOOH dissolution and the subsequent release of Fe–P; conversely, high Eh results in Fe^2+^ oxidation into Fe^3+^ and facilitates Fe–P formation [20]. Numerous studies have focused either on the reduction in P concentration (by submerged macrophyte restoration) [4,5,6] or the passivation of P (by microbial inoculation) in sediments [7,13,14,15]. However, few studies have explored the synergistic effects of submerged macrophyte restoration and microbial inoculation on Fe–P formation and P retention in sediments.

In the present study, sediments were sampled from a highly eutrophic freshwater lake and subjected to combinations of *V. natans* planting and IOB inoculation treatments. The objective was to explore the synergistic effects of *V. natans* and IOB on Fe–P distribution and to reveal the mechanisms of Fe–P formation in eutrophic lake sediments. Our hypotheses were as follows: (1) synergistic interactions between *V. natans* growth and IOB activity enhance Fe–P formation and reduce P concentrations in sediments; and (2) the introduction of *V. natans* and IOB alters the structure of sediment bacterial communities that mediate P retention. The results of the present study provide scientific evidence that could facilitate the control of internal P loading from freshwater sediments based on an integrated and environmentally friendly technology.

## 2. Materials and Methods

### 2.1. Experimental Materials

We selected *V. natans*, a widely distributed submersed macrophyte, as the experimental material. *V. natans* seedlings were collected from Qinhu Lake (32°37′ N, 120°09′ E) in Taizhou (Jiangsu Province, China), a eutrophic lake under ecological restoration. After 15 days of acclimation in a simulated lake system, the seedlings were rinsed thoroughly with distilled water.

**Figure 1:**
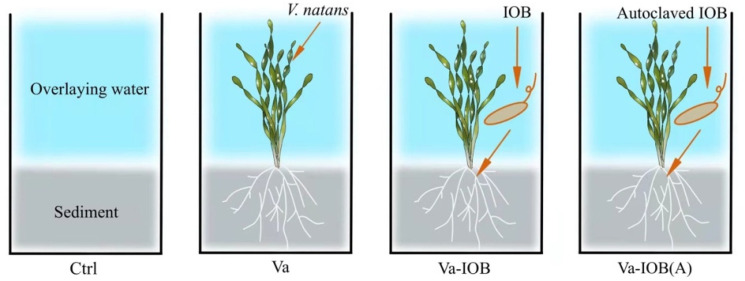
Overview of the experimental setup and treatment arrangement. Ctrl, no *V. natans* planting or iron-oxidizing bacteria (IOB) inoculation; Va, planting *V. natans* without IOB inoculation; Va-IOB, planting *V. natans* with IOB inoculation; Va-IOB(A), planting *V. natans* with autoclaved IOB inoculation.

Glass tanks (30 cm high × 15 cm wide × 15 cm long) were used to simulate the lake system (Figure 1), using sediment and water samples collected from the sampling site of *V. natans* seedlings. On 9 September 2020, surface water was collected from the lake at a depth of 0–20 cm, and 20 replicate sediment cores (15 cm inner diameter × 30 cm long) were collected using a gravity corer (15 cm diameter × 50 cm long; Rigo Co., Saitama, Japan). All of the sediment samples were mixed thoroughly with appropriate amounts of lake water and filtered through a 0.2 cm sieve to remove coarse debris. After homogenization, the sediment was transferred into glass tanks followed by lake water, yielding a 10 cm layer of sediment and 15 cm of overlying water.

An improved gradient tube method was used to obtain IOB [21]. First, the inoculum was prepared by vortexing a mixture containing 10 mL of sterilized deionized water and 1 g of sediment sample from Qinhu Lake for approximately 30 min. Subsequently, an opposite gradient of oxygen and Fe^2+^ (as FeS) was generated in glass bottles (50 mm high × 16 mm diameter). The inoculum (100 μL each) was inoculated along the vertical axis of the gradient tubes at serial dilutions (10^−1^ to 10^−5^) to allow for the growth of IOB at the artificial oxic–anoxic interface. The gradient tubes were incubated at 25 °C in the dark. On the second day after the formation of solid-phase Fe oxide bands (1st generation), approximately 10% of the band was extracted from the gradient tubes with the highest dilution of inoculum and then inoculated into a fresh gradient tube. The process was repeated over a two-week period for six generations to enrich IOB.

### 2.2. Experimental Design

There were four experimental treatments (Figure 1), namely no *V. natans* planting or IOB inoculation (control), *V. natans* planting without IOB inoculation (Va), *V. natans* planting with IOB inoculation (Va-IOB), and *V. natans* planting with autoclaved IOB inoculation (Va-IOB[A]). Each treatment had four replicates (glass tanks).

Acclimated *V. natans* seedlings were transplanted into glass tanks on 12 September 2020. With the exception of the control treatment, three plants were transplanted to the center of each tank after filling with water. All tanks were set up in an open room with a transparent roof and were exposed to natural sunlight. During the experimental period (12 September 2020–25 December 2020), the indoor temperature was maintained at 18 °C on average. An appropriate amount of sterilized deionized water was added to each tank once every three days to maintain the initial water level.

IOB inoculation was conducted in the early rapid growth stage of *V. natans* (four weeks after transplanting). In brief, IOB-containing bands were collected from one-week-old culture in gradient tubes with a sterile syringe (20 mL), thoroughly mixed, and divided into two portions. One portion was used directly for Va-IOB treatment, while the other portion was autoclaved at 121 °C for 30 min to kill the bacteria and then used for Va-IOB(A) treatment. The colloidal solution containing IOB (autoclaved or not, 5 mL each) was inoculated into the sediment layer in glass tanks at a 5 cm depth through a sterilized syringe (10 mL).

### 2.3. Sediment Physicochemical Analysis

Sediment samples (30 g each) were collected from each glass tank using a custom-made columnar mud collector (1 cm diameter) on the transplanting day (12 September 2020), rapid growth stage (28 October 2020), and mature stage of *V. natans* (25 December 2020). Each sediment sample was immediately divided into three subsamples. One subsample was air-dried and passed through a 0.15 mm sieve for use in the determination of conventional physicochemical properties. The second subsample was vacuum freeze-dried at –50 °C for use in the determination of Fe content. The third subsample was immediately stored in a refrigerator at –80 °C until use for bacterial community analysis.

Sediment pH and Eh were measured in situ using a portable pH meter (PHB-4; Leici, Shanghai, China) and an Eh meter (SX712; Ruisun, Chengdu, China), respectively [9]. Organic matter (OM) content in sediments was determined based on wet combustion with K_2_Cr_2_O_7_, and measured through titration with (NH_4_)_2_Fe(SO_4_)_2_·6H_2_O [22]. The dissolved Fe^2+^ and Fe^3+^ concentrations in sediments were measured using *o*-phenanthroline spectrophotometry [23]. The total P (TP) concentration in sediments was determined using the ascorbic acid method [24]. Fe–P was graded according to the harmonized protocol of the Standards, Measurements, and Testing Programme of the European Commission [17] and was determined by molybdenum-antimony anti-spectrophotometry [24].

### 2.4. Bacterial Community Analysis

Total genomic DNA was extracted from 0.5 g frozen sediment samples using a PowerSoil DNA extraction kit (Qiagen Inc., Valencia, CA, USA) according to the manufacturer’s instructions. Universal primers 338F (5′-ACTCCTACGGGAGGCAGCAG-3′) and 806R (5′-GGACTACHVGGGTWTCTAAT-3′) were used to amplify the V3–V4 region of the bacterial 16S rRNA gene [25]. PCR reactions were carried out in a total volume of 25 µL on an Applied Biosystems MiniAmp Plus Thermal Cycler (Thermo Fisher Scientific, Waltham, MA, USA). After purification and quantification, the PCR products were sequenced on an Illumina MiSeq platform (Illumina, San Diego, CA, USA) using a 468 bp paired-end protocol.

### 2.5. Data Analysis

Bioinformatics analysis of high-throughput sequencing data was performed on the Majorbio I-Sanger Cloud Platform (http://www.i-sanger.com; accessed on 11 May 2021). The effective sequences were clustered into operational taxonomic units (OTUs) at 97% sequence similarity [26]. The species of each OTU were annotated using the bacterial 16S rRNA Silva database (Release132, http://www.arb-silva.de; accessed on 14 May 2021) to obtain bacterial taxonomic information of each sample and the community species composition at each classification level. The OTU data were normalized, and the bacterial community diversity of each sample was analyzed in terms of observed number of species (Sobs), abundance-based coverage estimator (ACE), and Shannon diversity index, using Mothur [27].

Hierarchical clustering analysis of bacterial communities at the OTU level was performed in PRIMER 5 (PRIMER-E Limited, UK) based on the unweighted pair group method with arithmetic mean (UPGMA) method. Key bacterial taxa associated with Fe redox transformation were identified by comparing the results with data available in the National Center for Biotechnology Information (NCBI) database (https://www.ncbi.nlm.nih.gov; accessed on 20 November 2021) and previously published articles [28,29]. Functional annotation of prokaryotic taxa (FAPROTAX) was carried out (http://www.zoology.ubc.ca/louca/FAPROTAX/; accessed on 28 December 2021) to predict the potential functions of bacterial communities based on the 16S rRNA gene data [30].

Statistical analyses were performed in IBM SPSS Statistics 26 (IBM Corp., Armonk, NY, USA). Differences in sediment physicochemical properties, bacterial α-diversity indexes, and dominant taxa abundance between samples were determined using one-way analysis of variance and Tukey’s multiple comparison tests. *p* < 0.05 was considered an indication of statistical significance. Redundancy analysis (RDA) was carried out using Canoco 5.02 (Microcomputer Power, Ithaca, NY, USA) to determine the environmental variables that best explained variation in bacterial community structure in sediments treated with *V. natans* planting and IOB inoculation.

## 3. Results

### 3.1. Sediment pH, Organic Matter Content, and Redox Potential

**Figure 2:**
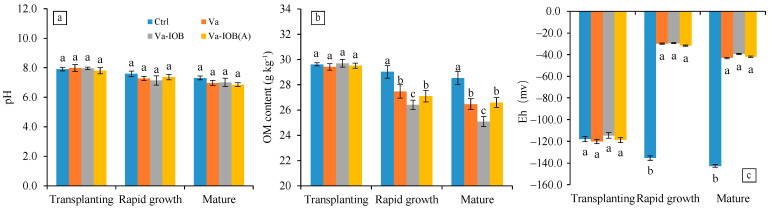
(**a**) pH, (**b**) organic matter (OM) content, and (**c**) redox potential (Eh) trends in sediments under different treatments during *V. natans* growth. Values are means ± standard deviation. For each stage, different lowercase letters above or below bars indicate significant differences at the 0.05 significance level by Tukey’s test.

The pH in sediments of all treatments decreased from the transplanting to rapid growth and then mature stages of *V. natans*, with mean values of 7.92, 7.34, and 7.01, respectively (Figure 2a). In the later growth stages, pH exhibited decreasing trends under the three *V. natans* treatments (Va, Va-IOB, and Va-IOB[A]); however, there was no significant difference with the pH of the control treatment.

The OM contents in sediments of all treatments also decreased from the transplanting stage to the mature stage (Figure 2b; *p* < 0.05). Sediment OM contents of the *V. natans* treatments were all significantly lower than that of the control treatment. In particular, the OM contents under the Va-IOB treatment were the lowest, in both the rapid growth and mature stages (*p* < 0.05).

Eh in sediments of the control treatment decreased significantly in the rapid growth and mature stages when compared with that in the transplanting stage (*p* < 0.05; Figure 2c). However, after *V. natans* planting, Eh increased significantly from the transplanting to rapid growth stage (*p* < 0.05), followed by a slight drop thereafter. No significant difference in Eh was observed between the treatments with and without IOB inoculation.

### 3.2. Sediment Iron, Phosphorus, and Iron-Bound Phosphorus Concentrations

**Figure 3:**
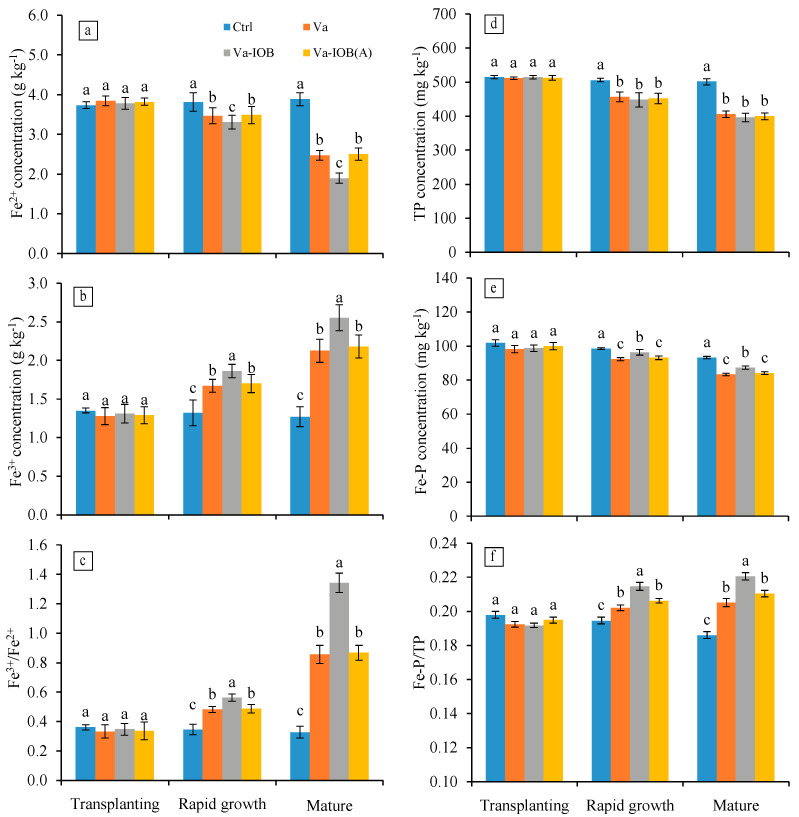
Iron and phosphorus concentrations in sediments under different treatments. (**a**) Dissolved Fe^2+^; (**b**) dissolved Fe^3+^; (**c**) ratios between Fe^3+^ and Fe^2+^; (**d**) total phosphorus (TP); (**e**) iron-bound phosphorus (Fe–P); and (**f**) proportions of Fe–P in TP (Fe–P/TP). Values are means ± standard deviation. For each stage, different lowercase letters above bars indicate significant differences at the 0.05 significance level by Tukey’s test.

With the exception of the control treatment, Fe^2+^ concentrations in sediments decreased from the transplanting stage to the mature stage (*p* < 0.05; Figure 3a). In the rapid growth and mature stages, Fe^2+^ concentrations under the Va-IOB treatment (3.31 and 1.90 mg kg^−1^, respectively) were significantly lower than those under the Va and Va-IOB(A) treatments (*p* < 0.05). Conversely, Fe^3+^ concentrations in sediments treated with *V. natans* increased significantly from transplantation to maturity, with the highest values observed under the Va-IOB treatment in the later growth stages (*p* < 0.05; Figure 3b). In the rapid growth and mature stages, the ratios between Fe^3+^ and Fe^2+^ concentrations increased markedly in sediments treated with *V. natans*, especially under the Va-IOB treatment (*p* < 0.05; Figure 3c).

Compared with the control treatment, TP concentrations in sediments treated with *V. natans* decreased significantly during the rapid growth and mature stages (*p* < 0.05; Figure 3d). However, there were no significant differences in TP concentrations among the Va, Va-IOB, and Va-IOB(A) treatments. Similarly, Fe–P concentrations had no significant differences between the Va and Va-IOB(A) treatments in the later growth stages, with mean values of 92.75 and 83.65 mg kg^−1^, respectively, lower than that of the Va-IOB treatment (Figure 3e). During the later growth stages, the proportions of Fe–P in TP significantly increased in sediments treated with *V. natans*, and the highest value was observed under the Va-IOB treatment (*p* < 0.05; Figure 3f).

### 3.3. Sediment Bacterial Diversity and Community Composition

The calculated α-diversity indexes of bacterial communities in sediment samples under different treatments are listed in Table 1. The Sobs and ACE indexes represent bacterial species richness, while the Shannon index represents bacterial species evenness; higher values of the α-diversity indexes suggest greater bacterial richness and lower heterogeneity. Overall, the mean coverage among all the samples was 97.3% (range: 95.1–98.7%). From the transplanting to mature stages of *V. natans*, the Sobs and ACE indexes decreased under the control treatment but increased under the other treatments with *V. natans*. The patterns of the Shannon index among different stages and treatments were similar to those of the species richness indexes. In the rapid growth and mature stages, the lowest Sobs, ACE, and Shannon index values were observed under the control treatment, while the highest ACE and Shannon index values were observed under the Va-IOB treatment.

**Figure 4:**
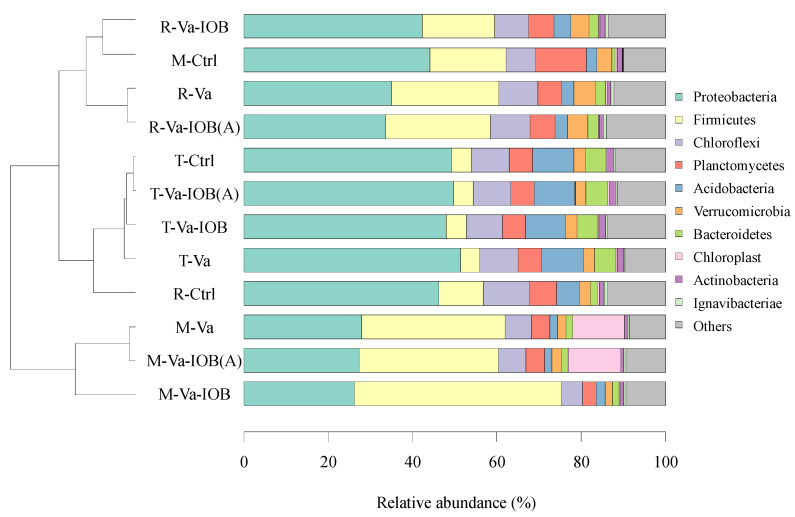
Clustering analysis of bacterial communities at the operational taxanomic unit (OTU) level (left) and bacterial community composition at the phylum level (right) in sediments under different treatments. T-, R-, and M- denote the transplanting, rapid growth, and mature stages of *V. natans*, respectively. Hierarchical clustering analysis was performed using unweighted pair group method with arithmetic mean (UPGMA) based on unweighted UniFrac distances from taxa tables and OTU number. The top 10 most abundant bacterial phyla are shown.

Based on the results of unweighted UniFrac distance analysis, sediment bacteria were clustered into three groups (*p* < 0.05; Figure 4). Excluding the control, all samples were clearly separated in groups corresponding to *V. natans* growth stages. Group I contained Va, Va-IOB, and Va-IOB(A) samples of the rapid growth stage and control samples of the mature stage; group II contained the control, Va, Va-IOB, and Va-IOB(A) samples of the transplanting stage and control samples of the rapid growth stage; and group III contained Va, Va-IOB, and Va-IOB(A) samples of the mature stage. Among the retrieved OTUs, 86.0–91.5% were classified among the top 10 most abundant bacterial phyla. The most abundant phylum was Proteobacteria in group I (38.7% of the total sequences), Proteobacteria in group II (48.9%), and Firmicutes in group III (38.7%). From transplanting to maturity, the relative abundance of Proteobacteria decreased substantially, while an increasing trend was observed for Firmicutes in all treatments. Compared with the control treatment, a higher relative abundance of Firmicutes was observed under the *V. natans* treatments (Va, Va-IOB, and Va-IOB[A]) in both the rapid growth and mature stages.

**Figure 5:**
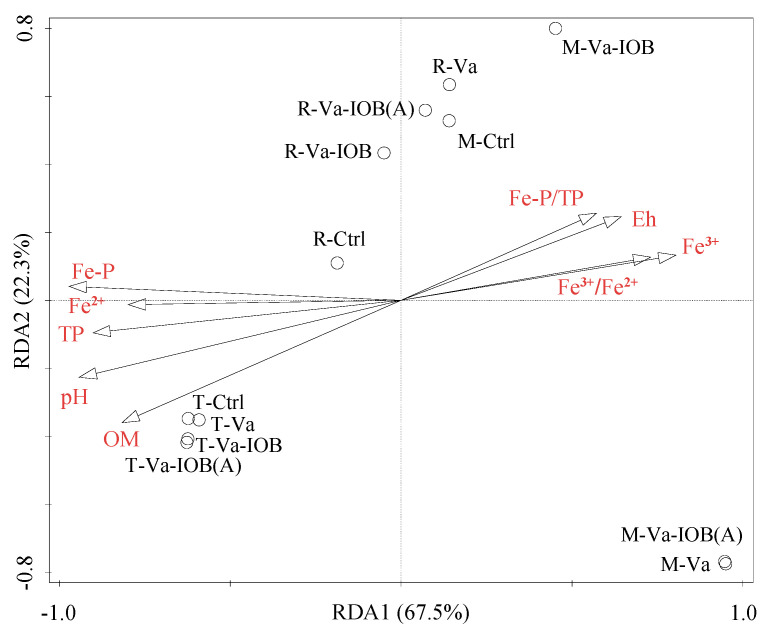
Redundancy analysis (RDA) ordination bi-plot showing the relationships between bacterial community structure (phylum level) and sediment environmental variables. OM, organic matter; Eh, redox potential; TP, total phosphorus; Fe–P, iron-bound phosphorus. Black circles represent treatments at different *V. natans* growth stages: T, transplanting; R, rapid growth; M, mature.

RDA was performed to explore the correlations between the relative abundances of bacterial phyla and sediment physicochemical properties. The model attributed 94.8% of the variance to phyla-environment correlations (Figure 5). The first and second axes accounted for 67.5% and 22.3% of the variation, respectively. In addition, Fe–P, TP, pH, and OM were the key factors significantly influencing bacterial community structure (*p* < 0.05).

### 3.4. Bacterial Taxa and Functional Genes Related to Iron Oxidation and Reduction

**Figure 6:**
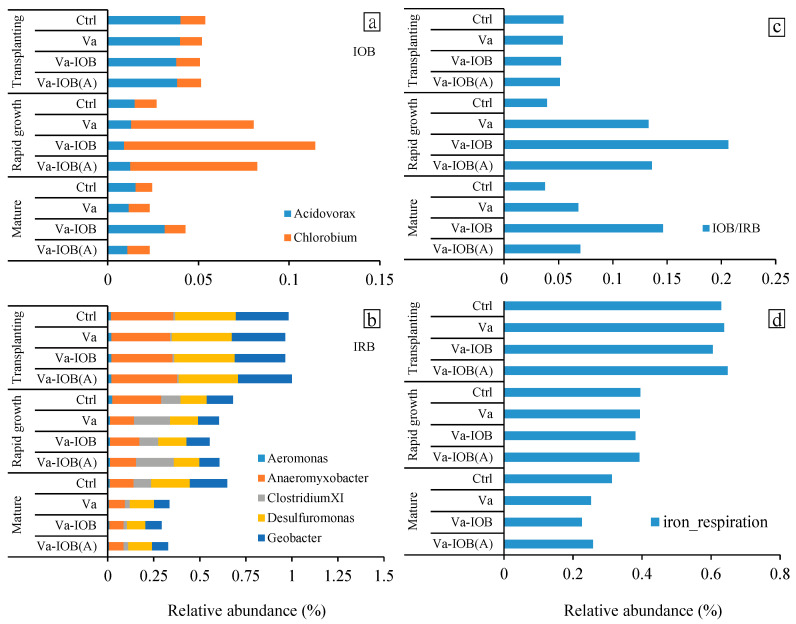
Key bacterial genera related to (**a**) iron oxidation and (**b**) iron reduction, (**c**) the ratio between iron-oxidizing bacteria (IOB) and iron-reducing bacteria (IRB), and (**d**) FAPROTAX-based functional prediction of sediment bacteria related to iron respiration under different treatments.

The dominant IOB at the genus level in all treatments included *Acidovorax* and *Chlorobium*, especially in the rapid growth stage. The relative abundances of IOB increased markedly from the transplanting to rapid growth stage and then decreased toward the mature stage (Figure 6a). In the later growth stages, the highest relative abundance of IOB was observed under the Va-IOB treatment. In contrast, the relative abundances of Fe^3+^-reducing bacteria (IRB) decreased substantially from transplantation to maturity (Figure 6b). In the rapid growth and mature stages, the relative abundances of IRB under the Va-IOB treatment were lower than those of other treatments. In addition, the IOB/IRB ratios in sediments treated with *V. natans* considerably increased in the later growth stages, and the highest ratio was observed under the Va-IOB treatment (Figure 6c).

Potentially related functions among bacterial communities were predicted using FAPROTAX 1.1, and the relative contributions of each functional bacterial group were explored (Figure 6d). The relative abundances of genes related to Fe reduction decreased considerably from transplantation to maturity. In particular, the relative abundances of Fe reduction-related genes were distinctively lower under the Va-IOB treatment compared with the other treatments at maturity. However, key ecological functions related to Fe oxidation were not predicted in any treatments.

## 4. Discussion

### 4.1. Synergistic Interactions of V. natans and Iron-Oxidizing Bacteria Enhance Iron-Bound Phosphorus Formation in Sediments

In the present study, we investigated the combined effects of *V. natans* growth and IOB inoculation on internal P loading from eutrophic lake sediments. We observed that TP concentrations in sediments decreased remarkably after the planting of *V. natans* with and without IOB inoculation. The results indicate that *V. natans* could control internal P loading by absorbing P from sediments. Our finding is consistent with the results of previous studies where only treatments with submerged macrophyte plantations could control internal P loading [4,5,6]. In another study, compared with the unplanted control, TP concentrations in sediments planted with *V. natans* and *Ceratophyllum demersum* decreased by 10.7% and 9.9%, respectively [31].

P migration in shallow eutrophic lake sediments is mainly regulated by redox transformation of Fe [32]. On the one hand, restoration of submerged macrophytes has the potential to increase sediment Eh and to enhance the oxidation of Fe^2+^ to Fe^3+^, leading to Fe–P formation and ultimately facilitating sediment P retention [33]. On the other hand, labile root exudates from submerged macrophytes could activate anaerobic organic matter-degrading bacteria, which might participate in biological reduction of Fe^3+^ or provide substrates for a chemical reduction of Fe^3+^, resulting in an increase in P release [15]. Here, planting *V. natans* with and without IOB inoculation enhanced Fe–P formation and increased the proportions of Fe–P in TP in sediments, indicating that Fe oxidation, rather than Fe reduction, is the dominant process.

In addition to chemical Fe oxidation, bacterially mediated Fe oxidation considerably enhanced Fe–P formation in the sediments. The biological effect was demonstrated by increases in the concentrations of Fe–P and proportions of Fe–P in TP under the Va-IOB treatment compared with the Va and Va-IOB(A) treatments. Multiple factors including OM, Eh, and dissolved oxygen influence the dynamics of IOB and IRB [34]. A previous study reported that higher Eh conditions are favorable for bacterially mediated Fe oxidation; the resulting FeOOH colloids can strongly absorb PO_4_^3−^ and can enhance Fe–P formation [35]. Similarly, we detected relatively high Eh, Fe^3+^ concentrations, and Fe^3+^/Fe^2+^ ratios under the Va-IOB treatment. This was probably caused by rapid Fe redox transformations mediated by the introduction of the IOB consortium, which thus enhanced the formation of Fe-bound P precipitates [36]. In summary, the results indicate that the synergistic interactions between *V. natans* and IOB enhanced Fe–P formation in sediments.

### 4.2. Synergistic Interactions of V. natans and Iron-Oxidizing Bacteria Increase Diversity of Sediment Bacterial Community

In the present study, the *V. natans* planting treatments with and without IOB inoculation increased the α-diversity of bacterial communities in sediments, especially in the rapid growth and mature stages. Such improvements in bacterial diversity could be attributed to lower OM contents in sediments planted with *V. natans*. Similarly, Lin et al. [4] observed that bacterial community diversity in macrophyte-treated sediments was higher than that in unplanted controls. Another study reported a negative correlation between bacterial community diversity and OM content in sediments at seven sampling sites in Taihu Lake, China [37]. Higher OM concentrations may favor the growth of dominant bacterial populations, which in turn inhibits the growth of non-dominant bacteria [38].

The results of clustering analysis at the OTU level indicated that transplantation and growth of *V. natans* plants distinctively altered bacterial community structure in sediments. *V. natans* planting with and without IOB inoculation led to the enrichment of Firmicutes in the rapid growth and mature stages. The phylum Firmicutes contains a variety of bacteria capable of degrading and transforming organic material, thereby enhancing the release of mineral elements [39]. Therefore, the higher relative abundance of Firmicutes in sediments planted with *V. natans* (without additional nutrient input) might enhance OM decomposition and provide nutrients for *V. natans* growth.

### 4.3. Key Taxa and Predicted Functions of Bacteria Associated with Phosphorus Retention in Sediments

Although environmental physicochemical factors play a key role in Fe redox activity, an increasing number of studies report that some bacterial groups exert major influence in the transition between Fe^2+^ and Fe^3+^ [15,40]. Numerous bacterial groups are capable of Fe reduction or oxidation in microaerobic and anaerobic environments [40]. The introduction of IOB and associated Fe oxides clearly increased the proportions of IOB in sediments during the rapid growth and mature stages of *V. natans*. This study identified the key bacterial genera associated with Fe redox transformation in sediments. Under all treatments, *Acidovorax* and *Chlorobium* were observed as dominant IOB. Members of *Acidovorax*, such as strains BoFeN1 and 2AN, are neutrophilic, nitrate (NO_3_^−^)-reducing, and Fe^2+^-oxidizing bacteria in anaerobic environments [41,42]. Therefore, NO_3_^−^ reduction would also play a role in Fe oxidation in the simulated lake system. In addition, our previous field study showed that N metabolism genes accounted for a substantial portion of genes related to energy metabolism in shallow lake sediments [36]. However, none of the bacterial groups in all treatments was predicted to have potential Fe-oxidizing genes. These bacteria might be omitted in predictions of Fe oxidation function using FAPROTAX 1.1. A previous study also indicated that the abundance of traditional IOB was substantially low in all sediment samples, and they were almost absent in samples with OM addition [17].

Some bacterial groups with sulfide oxidation or sulfate reduction capacity influence the formation of Fe redox species through the transformation of sulfide [15]. In the present study, the trends in the relative abundances of IOB among different treatments were similar to those of sulfide-oxidizing bacteria (Figure S1a), while the relative abundances of IRB and sulfate-reducing bacteria (Figure S1b) shared similar trends. Furthermore, the relative abundances of predicted functions, including sulfur (S) oxidation and N reduction, were higher in sediments planted with *V. natans*, especially under the Va-IOB treatment (Figure S2a). Such trends were similar to those of IOB, while the trends in the relative abundances of predicted functions associated with Fe^3+^ reduction (Figure S2b) were similar to those of IRB. Sulfide could simultaneously reduce Fe^3+^ and form FeS; hence, it reduces the availability of Fe to bind P in both ferric and ferrous solids [15]. Given the high portion of sulfate-reducing bacteria (e.g., Desulfobacteraceae) in the sediment samples, the contribution of these microorganisms to Fe–P formation could not be neglected.

Overall, the results of the present study indicate that bacterially mediated Fe redox reactions play essential roles in the retention of P in sediments with the presence of *V. natans*. That P retention in sediments is synergistically controlled by Fe oxidation, S oxidation, and NO_3_^−^ reduction. The underlying microbiological mechanisms of such synergistic interactions still require further investigations.

## 5. Conclusions

This study analyzed the interactive effects of *V. natans* planting and IOB inoculation on Fe–P formation in eutrophic lake sediments. *V. natans* growth decreased OM, Fe^2+^, and TP concentrations; increased Fe^3+^ and Fe–P concentrations; and improved bacterial diversity in sediments. The effects of *V. natans* were dependent on plant growth stage intensified by IOB inoculation. Moreover, *V. natans* and IOB introduction increased the relative abundances of IOB genera, such as *Acidovorax* and *Chlorobium*, especially in the rapid growth stage of *V. natans*. The results confirm our hypothesis that synergistic interactions of *V. natans* growth and IOB activity enhance Fe–P formation in eutrophic lake sediments through the modification of sediment physicochemical conditions and associated bacterial community structure. Overall, the coupled *V. natans*–IOB system shows great potential as an in situ remediation technology for controlling internal P loading from freshwater sediments. Our study was conducted under simulated conditions; therefore, the results must be interpreted with caution. Field experiments should be carried out to determine the optimal planting density of *V. natans* and inoculum level of IOB. Further studies on the key environmental factors and microbial taxa that influence the remediation process are also required to elucidate the microbiological mechanisms of IOB-mediated P retention in eutrophic sediments.

## Figures and Tables

**Table 1:** α-diversity of sediment bacterial communities evaluated based on operational taxonomic units (OTUs) clustered at the 97% similarity level.

Stage	Treatment	Observed Number of Species	Abundance-Based Coverage Estimator	Shannon Index	Coverage
Transplanting	Ctrl	1259.43 a	1409.15 a	5.27 a	0.980
	Va	1248.37 a	1418.30 a	5.32 a	0.966
	Va-IOB	1251.13 a	1397.05 a	5.24 a	0.987
	Va-IOB(A)	1251.40 a	1398.93 a	5.26 a	0.967
Rapid growth	Ctrl	1190.53 b	1335.38 c	5.04 c	0.980
	Va	1291.61 a	1456.95 b	5.48 b	0.951
	Va-IOB	1335.27 a	1481.73 a	6.06 a	0.958
	Va-IOB(A)	1284.31 a	1451.00 b	5.45 b	0.973
Mature	Ctrl	1112.28 b	1276.74 c	4.97 c	0.980
	Va	1294.71 a	1459.42 b	6.18 b	0.977
	Va-IOB	1319.32 a	1513.28 a	6.27 a	0.983
	Va-IOB(A)	1296.41 a	1452.00 b	6.11 b	0.979

Note: Ctrl, no *V. natans* planting or iron-oxidizing bacteria (IOB) inoculation; Va, planting *V. natans* without IOB inoculation; Va-IOB, planting *V. natans* with IOB inoculation; Va-IOB(A), planting *V. natans* with autoclaved IOB inoculation. For each stage, different lowercase letters in the same column indicate significant differences among the treatments based on Tukey’s test (*p* < 0.05).

## Data Availability

The data presented in this study are available from the corresponding author upon request.

## Supplementary Materials

The following are available online at https://www.mdpi.com/article/10.3390/microorganisms10020413/s1, Figure S1: Key bacterial genera related to (a) sulfide oxidation and (b) sulfate reduction in sediments under different treatments. Figure S2: Functional predictions of sediment bacteria related to (a) iron oxidation and (b) iron reduction by FAPROTAX.
