# Supplementary material for: Interactions of Vallisneria natans and Iron-Oxidizing Bacteria Enhance Iron-Bound Phosphorus Formation in Eutrophic Lake Sediments
Source: Microorganisms. 2022 Feb 11;10(2):413. doi: 10.3390/microorganisms10020413 (PMC8879316; doi:10.3390/microorganisms10020413)
Supplement: Supplementary file 1 [file microorganisms-10-00413-s001.zip › microorganisms-1554601-supplementary.pdf]

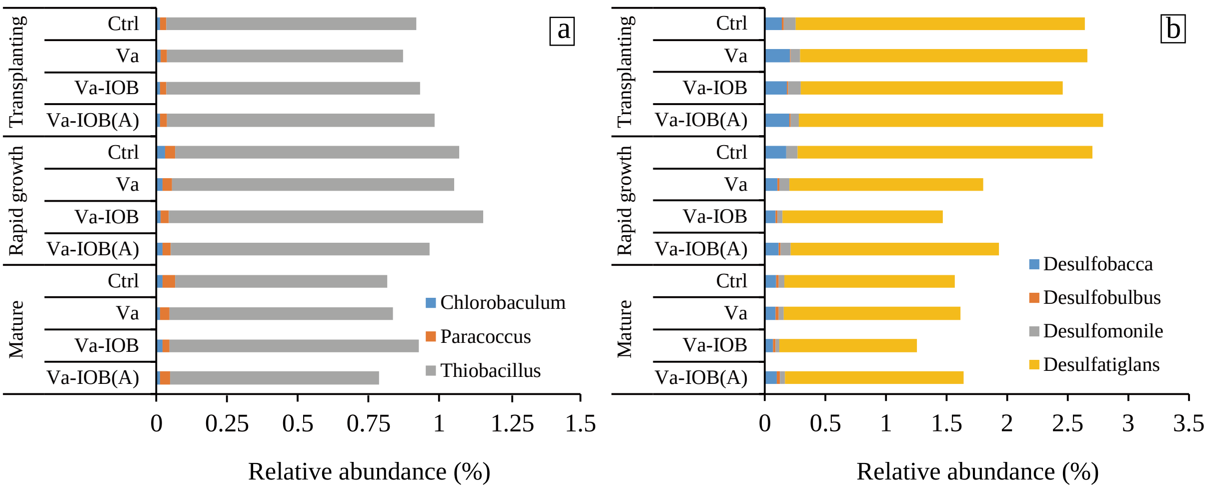

Figure S1. Key bacterial genera related to (**a**) sulfide oxidation and (**b**) sulfide reduction in sediments under different treatments. Ctrl, no *V. natans* planting or iron-oxidizing bacteria (IOB) inoculation; Va, planting *V. natans* without IOB inoculation; Va-IOB, planting *V. natans* with IOB inoculation; Va-IOB(A), planting *V. natans* with autoclaved IOB inoculation.

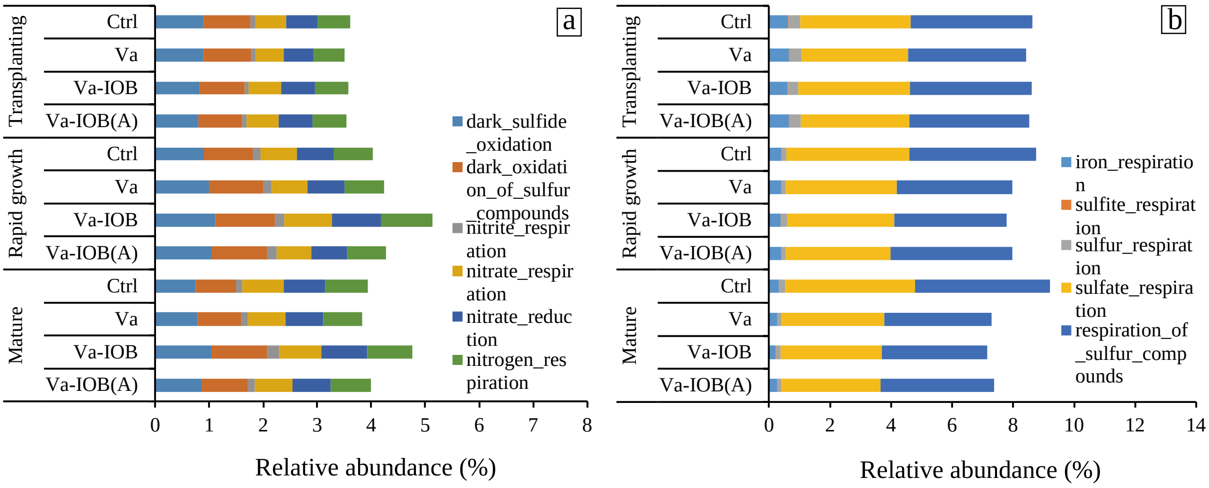

Figure S2. Functional predictions of sediment bacteria related to (**a**) iron oxidation and (b) iron reduction by FAPROTAX. Ctrl, no *V. natans* planting or iron-oxidizing bacteria (IOB) inoculation; Va, planting *V. natans* without IOB inoculation; Va-IOB, planting *V. natans* with IOB inoculation; Va-IOB(A), planting *V. natans* with autoclaved IOB inoculation.
